# Biodegradation of thiocyanate by a native groundwater microbial consortium

**DOI:** 10.7717/peerj.6498

**Published:** 2019-03-26

**Authors:** Liam P. Spurr, Mathew P. Watts, Han M. Gan, John W. Moreau

**Affiliations:** 1 School of Earth Sciences, University of Melbourne, Parkville, Australia; 2 School of Life and Environmental Sciences, Deakin University, Waurn Ponds, Australia

**Keywords:** Bioremediation, Thiocyanate, Mining, Contamination, Microorganisms, Groundwater, Geomicrobiology, Sulphurs-oxidation

## Abstract

Gold ore processing typically generates large amounts of thiocyanate (SCN^−^)-contaminated effluent. When this effluent is stored in unlined tailings dams, contamination of the underlying aquifer can occur. The potential for bioremediation of SCN^−^-contaminated groundwater, either in situ or ex situ, remains largely unexplored. This study aimed to enrich and characterise SCN^−^-degrading microorganisms from mining-contaminated groundwater under a range of culturing conditions. Mildly acidic and suboxic groundwater, containing ∼135 mg L^−1^ SCN^−^, was collected from an aquifer below an unlined tailings dam. An SCN^−^-degrading consortium was enriched from contaminated groundwater using combinatory amendments of air, glucose and phosphate. Biodegradation occurred in all oxic cultures, except with the sole addition of glucose, but was inhibited by NH_4_^+^ and did not occur under anoxic conditions. The SCN^−^-degrading consortium was characterised using 16S and 18S rRNA gene sequencing, identifying a variety of heterotrophic taxa in addition to sulphur-oxidising bacteria. Interestingly, few recognised SCN^−^-degrading taxa were identified in significant abundance. These results provide both proof-of-concept and the required conditions for biostimulation of SCN^−^ degradation in groundwater by native aquifer microorganisms.

## Introduction

Thiocyanate (SCN^−^) is a toxic contaminant in industrial wastewater streams associated with gold mining (Stott et al., 2001; Akcil, 2003; Kenova, Kormienko & Drozdov, 2010), steel production (Lay-Son & Drakides, 2008), photofinishing (Shukla et al., 2004), electroplating (Aguirre et al., 2010), herbicide and insecticide production (Hughes, 1975) and coal coking (Dash, Gaur & Balomajumder, 2009; Gould et al., 2012). In gold ore processing, SCN^−^ is generated through reaction of cyanide (CN^−^) with sulphide minerals and other intermediate valence sulphur species (Akcil, 2003). Most mine operators promote this reaction, as CN^−^ is even more toxic than SCN^−^ (Ingles & Scott, 1987). However, SCN^−^ remains an undesirable end-product that must be removed for safe storage or disposal of waste water.

The waste products of gold ore processing are typically stored for indefinite time periods within large tailings storage facilities (TSFs). Many TSFs were historically unlined, such that tailings-derived SCN^−^-leachate flows directly into the underlying water table. Although hydrologic recirculation of SCN^−^-contaminated groundwater to the TSF has been used to retard SCN^−^ migration, this strategy is unsustainable both as an environmental or an economical long-term solution for aquifer remediation.

Thiocyanate remediation technologies have drawn recent interest, due to the environmental stability (Gould et al., 2012) and potential toxicity of SCN^−^ to aquatic life, with an LC_50_ for *Daphnia magna* of 0.55–33.47 mg L^−1^ (Watson & Maly, 1987). Abiotic SCN^−^ remediation techniques typically involve chemical oxidation (e.g. SO_2_/air, peroxide and Caro’s acid) (Wilson & Harris, 1960; Breuer, Jeffrey & Meakin, 2011) or adsorption/separation methods (Aguirre et al., 2010). These approaches are often expensive to implement, and may produce sulphuric or nitric acid waste streams (Akcil, 2003; Dash, Gaur & Balomajumder, 2009). Bioremediation of SCN^−^ would present a more cost-effective alternative (Akcil, 2003), either via (1) stimulation of SCN^−^-degrading microorganisms within extracted groundwater, prior to re-injection into the contaminated aquifer; or (2) promotion of in situ biodegradation within the aquifer under ambient conditions. The former approach, likely implemented in the form of a bioreactor, has gained much attention for treating SCN^−^ containing effluent streams (Whitlock, 1990; Van Zyl, Harrison & Van Hille, 2011; Villemur et al., 2015), while in situ approaches remain largely unexplored.

Thiocyanate-degrading microorganisms occupy a diverse range of environments, including activated sludge (Van Zyl, Harrison & Van Hille, 2011), soils (Vu, Mu & Moreau, 2013) and soda lakes (Sorokin et al., 2004, 2014). SCN^−^-degraders can use SCN^−^ as a source of sulphur, carbon, nitrogen and energy (Sorokin et al., 2001; Ebbs, 2004; Grigor’eva et al., 2006), producing sulphate and ammonia, the latter of which can be reduced via denitrification to nitrogen gas. These microorganisms exhibit both heterotrophic (Vu, Mu & Moreau, 2013) and autotrophic metabolisms (Sorokin et al., 2001, 2004; Bezsudnova et al., 2007; Huddy et al., 2015, Watts et al., 2017), with the former typically using SCN^−^ as a nitrogen source and the latter oxidizing sulphur as an energy source. Much work has been done to understand the complex community interactions in engineered systems treating wastewater (Lee et al., 2008; Kantor et al., 2015, 2017). Most commercial SCN^−^ bioremediation systems use organic carbon amendments to treat contaminated slurry waste streams, while no previous studies have focussed on groundwater as inoculum and simple inorganic amendments.

In this study, we hypothesised that SCN^−^-contaminated groundwater could be enriched for microorganisms capable of biodegrading SCN^−^ if supplemented with a primary limiting nutrient: bioavailable phosphate. We experimentally determined the potential for SCN^−^ biodegradation by the groundwater microbiome, and characterised its diversity and phylogeny. Our approach involved (1) enrichment of SCN^−^-degrading microorganisms from contaminated groundwater, (2) culturing experiments involving amendments of dissolved organic carbon (DOC) (glucose), PO_4_^3−^ and NH_4_^+^; and (3) Illumina MiSeq sequencing of 16S and 18S rRNA genes from the SCN^−^-degrading microbial consortium. As SCN^−^ potentially provides carbon and nitrogen to microorganisms, we quantified the extent to which external amendments of NH_4_^+^ or DOC to groundwater impacted SCN^−^ biodegradation rates (Paruchuri, Shivaraman & Kumaran, 1990). We also measured the impact of a bioavailable PO_4_^3−^ amendment to groundwater-derived mixed cultures on SCN^−^-biodegradation rates, expanding on similar experiments in mine tailings water (Watts et al., 2017). The motivation for using groundwater-derived microbial communities was to explore the potential for bioremediating SCN^−^ in mining-impacted aquifers or extracted bore waters. Our results present new information and insights to increase efficiency and reduce costs for SCN^−^ treatment by the mining industry worldwide.

## Materials and Methods

### Groundwater sampling and storage

Mining-contaminated groundwater was extracted from a monitoring well located adjacent to a TSF at an operational gold mine in central Victoria, using a low flow pump. The well was screened in weathered granodiorite and schist, at a depth of 55 m below the surface. A sample of approximately 15 L was extracted, sealed and stored on ice until it was returned to the lab the next day, where it was refrigerated at 4 °C until use. The groundwater was used for enrichments within 3 days of sampling, while the remaining groundwater (with no trace metals or vitamins added) was used as a filter-sterilised medium for further culturing transfers, having been stored for up to 10 weeks in the dark at 4 °C by the end of the experiments. The chemistry of the groundwater is monitored frequently at the site and is typically moderately saline (TDS 11,425 ± 457), has a temperature of 15.2 °C ± 1.6, pH of 6.6–6.8, SCN^−^ concentrations of 500–1,000 mg L^−1^ and free CN^−^ concentrations of <0.03 mg L^−1^ (Table A1). We thank Stawell Gold Mine for access to the field site and historical monitoring data (no permit number issued).

### Groundwater geochemical analyses

At the time of sampling, a flow cell was used to determine the pH, E_H_ and DO measurements taken with a YSI Professional Plus™ multi-parameter meter with calibrated probes. The groundwater was also analysed by colorimetry for SCN^−^ Sörbo (1957) and NH_4_^+^ upon return to the laboratory, using the ferric-nitrate method (Eaton & Franson, 2005) and the salicylate-nitroprusside method (Baethgen & Alley, 1989), respectively. During laboratory-based experiments, pH was measured using a Thermo Orion 5 Star Plus™ Electrolyte Analyser and calibrated probes. Growth of the culture was monitored by tracking optical density at 600 nm (OD_600_). All colorimetric analyses were conducted using a Hach DR2800™ Portable Spectrophotometer with standard solutions.

### Aerobic and anaerobic enrichment culturing experiments

Groundwater was incubated under oxic and anoxic conditions, with various nutrient amendments, to enrich an SCN^−^-degrading culture. The oxic replicates were made by decanting groundwater (100 mL) under sterile conditions into triplicate autoclaved 250 mL conical flasks, sealed with a cotton wool bung and foil. Anoxic cultures were prepared by adding 30 mL of groundwater to 50 mL serum bottles sealed with rubber stoppers and aluminium crimps, and degassed using pressurised nitrogen gas. Both the oxic and anoxic cultures were further amended with additions of either 5 g L^−1^ DOC (as glucose) or 50 mg L^−1^ PO_4_^3−^ (as NaH_2_PO_4_), or both, alongside no-addition controls. All incubations were maintained in the dark on a rotary shaker at 30 °C and 120 rpm. The SCN^−^ concentration was monitored to determine if degradation was occurring; in cases where complete or near complete SCN^−^ removal was noted, this enrichment provided inoculum for further culturing using filter sterilised (0.22 μm filter) groundwater as the medium. The amendments that produced a stable SCN^−^ degrading culture upon further culturing were selected for further study.

Oxic cultures were sampled by extracting two mL with a sterile syringe in a biosafety hood to ensure sterility. Anoxic cultures were sampled by extracting two mL of the culture with a N_2_-degassed sterile syringe and needle, sampled through the rubber stopper. Half of the sample was passed through a 0.22 μm filter, while the other half was used for OD_600_ measurement prior to freezing at −20 °C. Samples for DNA sequencing were removed at late-log phase growth from microbial cultures stably degrading SCN^−^ after five transfers, and immediately frozen at −80 °C until thawing for DNA extraction.

### Culturing of a SCN^−^-degrading microbial consortium from groundwater

Amendments that resulted in a groundwater culture capable of SCN^−^ degradation after repeated culturing were further tested. All culturing after the initial enrichment phase was performed in sterilised 250 mL conical flasks, containing 100 mL of filter-sterilised ground water, stoppered using a cotton wool bung and foil, and with previously used nutrient amendments. For transfer to fresh medium, 10% v/v of the inoculum culture was sampled in late log phase of growth and incubated on a rotary shaker (or in a static incubator, in the case of anoxic cultures) at 30 °C and 120 rpm in the dark. Before subsequent testing, the culture was routinely re-cultured a minimum of five times to ensure a stable microbial community had developed. To determine the behaviour of the end-product, NH_4_^+^, an identical culturing experiment to those previously described was set-up and samples removed to monitor SCN^−^, OD_600_ and NH_4_^+^.

### Biodegradation of SCN^−^ in the presence of ammonium

A further experiment was set up to determine the impact of NH_4_^+^ on SCN^−^ biodegradation. This experiment used the re-cultured SCN^−^-degrading microbial community and was again performed using filter-sterilised (0.22 μm filter) groundwater from the same well. As with previous experiments, this was performed in triplicate 250 mL conical flasks, containing 100 mL of filter-sterilised groundwater, stoppered using a cotton wool bung and foil. The flasks were amended to low (no addition), moderate (10 mg L^−1^) and high (40 mg L^−1^) concentrations of NH_4_^+^ using a filter-sterilised (0.22 μm) concentrated (NH_4_)_2_SO_4_ solution, in addition to five g L^−1^ glucose and 50 mg L^−1^ PO_4_^3−^. An inoculum of the late log-phase culture was then added at 10% v/v concentration and incubated on a rotary shaker at 30 °C and 120 rpm in the dark.

### Whole community microbial DNA extraction and Illumina MiSeq 16S and 18S rRNA gene sequencing

The triplicate samples for microbial ecology analysis were firstly removed from the −80 °C freezer and thawed. The genomic DNA was then extracted with the PowerSoil DNA Isolation Kit (Mo Bio Laboratories, Inc. Carlsbad, CA, USA). The primers used for 16S and 18S rRNA gene sequencing consist of partial Illumina adapter at the 5′ end. The incorporation of the second-half of the Illumina adapter and dual-index barcode was performed in another round of PCR reaction (Illumina 16S Sequencing Protocol). The 16S and 18S rRNA gene amplicon sequencing (Caporaso et al., 2012) was performed using on Illumina MiSeq platform (Illumina, San Diego, CA, USA) located at the Monash University Malaysia Genomics Facility (2 × 250 bp run configuration).

The 16S rRNA gene was amplified by PCR using primers targeting the V3-V4 region of the 16S gene: Forward 5′-CCTACGGGNGGCWGCAG-3′ and Reverse 5′-GACTACHVGGGTATCTAATCC-3′ (Klindworth et al., 2013). High-fidelity PCR was performed on one μL of each DNA sample using 0.5 μL of Illumina-compatible universal primers, under the following thermal cycler conditions: initial denaturation step (98 °C for 30 s), followed by 25 cycles of denaturation (98 °C for 10 s), annealing (60 °C for 30 s) and extension (65 °C for 60 s), followed by a final extension step (65 °C for 120 s). The product was further purified with 20 μL of Ampure 0.8×, and washed with 200 μL of 80% ethanol and eluted in 50 μL for subsequent index ligation using Nextera XT Index primers i7 forward and i5 reverse Illumina adapters. The subsequent product was purified with 12 μL of Ampure 0.8×, washed with 200 μL of 80% ethanol, and eluted in 30 μL for sequencing.

The 18S rRNA gene from the genomic DNA samples was amplified using the forward primer 1391f 5′-GTACACACCGCCCGTC-3′, and the reverse primer EukBr 5′-AGACAGTGATCCTTCTGCAGGTTCACCTAC-3′ (Amaral-Zettler et al., 2009). PCR was performed with one μL of the DNA extract in the presence of 10 μM Illumina-compatible primer, under the following thermal cycler conditions; initial denaturation (98 °C for 30 s), followed by 25 repetitions of denaturation (98 °C for 10 s), annealing (65 °C for 60 s) and extension (65 °C for 120 s) and a final extension (65 °C for 120 s). This PCR product was purified with 25 μL of Ampure 1× and washed with 200 μL of 80% ethanol, then eluted in 40 μL in preparation for index ligation, using Nextera XT Index i7 forward primer Nextera XT Index i5 reverse primer. The product was purified with 10 μL of Ampure 1, washed with 200 μL of 80% ethanol and eluted in 30 μL for 18S sequencing.

### 16S and 18S rRNA gene sequence analysis

Prior to any bioinformatic processing, the raw 18S and 16S rRNA gene sequences were uploaded to the National Centre for Biotechnology Information’s (NCBI’s) sequence read archive, with the BioProject accession number PRJNA356784. Analysis of the 18S and 16S rRNA gene sequencing data was performed using the QIIME software package in order to determine the phylogenetic structure of the microbial community (Caporaso et al., 2010a). Bases with a phred quality score of <20, and reads that retained less than 75% of their original sequence length, were discarded. Forward and reverse reads of the 16S and 18S rRNA genes were joined at paired ends and aligned. The sequences were demultiplexed, filtered and processed through the QIIME software package (Caporaso et al., 2010a).

The 16S rRNA gene sequences were compared to those in the GreenGenes Bacterial and Archaeal 16S rRNA gene database (DeSantis et al., 2006) using BLAST (Altschul et al., 1990), picking operational taxonomic units (OTUs) at a 97% similarity cut-off. Representative sequences from each OTU were aligned using the PyNAST tool (Caporaso et al., 2010b), and chimeric sequences were identified and removed using ChimeraSlayer (Haas et al., 2011). The 18S rRNA gene sequences were assigned to OTUs via a de novo approach using USEARCH v5.2.236 (Edgar, 2010). Representative sequences from each OTU were checked for chimeric sequences, and these were removed using UCHIME v6.1.544 (Edgar et al., 2011). The resulting OTUs were assigned taxonomy by comparison to the SILVA 16S/18S rRNA gene database (SILVA 119, Quast et al., 2013) using Blastall v2.2.22. All OTUs that were assigned to prokaryotic taxa were then removed from the 18S rRNA gene dataset.

Operational taxonomic units representing >1% abundance were processed through the NCBI BLAST program and assigned taxonomies according to highest sequence similarity. The resulting BLAST assigned identities were compared to the taxonomic identities assigned by the GreenGenes and SILVA databases for the 16S rRNA (Table A2) and 18S rRNA sequencing (Table A3), respectively.

## Results

### Groundwater chemistry

The geochemical conditions of the groundwater at the time of sampling are presented in Table 1. The groundwater pH was slightly acidic and contained SCN^−^ in addition to a small concentration of NH_4_^+^. The prevailing redox conditions in the groundwater were reducing, with low oxygen levels.

**Table 1:** Basic groundwater chemistry data.

Groundwater chemistry	
SCN^−^ (mg L^−1^)	135 ± 1.73
NH_4_^+^ (mg L^−1^)	8.9 ± 1.9
pH	6.5 ± 0.03
Dissolved O_2_ (%)	2.0 ± 0.5
E_H_ (mV)	−21.6 ± 2.0
Conductivity (mS cm^−1^)	17.64 ± 0.1
T °C	16.3 ± 0.1

**Notes:**

Groundwater chemistry at the time of sampling. Errors are equal to 1 standard deviation within triplicate samples of the groundwater. Full groundwater chemistry (quarterly reports) is presented in Table A1.

### Groundwater enrichment culturing experiments

During the initial enrichment experiment, no SCN^−^ removal was noted in the absence of oxygen, regardless of nutrient amendment (see Table 2 for initial and final SCN^−^ concentrations). In the oxic enrichment experiments, SCN^−^ removal was observed in the absence of any nutrient amendment; however, upon inoculation of this culture into filter-sterilised groundwater, no SCN^−^ removal was observed. The sole addition of DOC or PO_4_^3−^ also resulted in SCN^−^ biodegradation in the initial enrichment, but when transferred into filter-sterilised groundwater, SCN^−^ degradation did not occur. The only condition to result in a SCN^−^ degrading culture, which was culturable in filter-sterilised groundwater, was the addition of DOC and PO_4_^3−^ in the presence of air. Initially, complete removal of SCN^−^ (from approximately 130 mg L^−1^) was achieved within 4 days, through combined addition of DOC and PO_4_^3−^. This culture was used for subsequent experiments.

**Table 2:** Enrichment experiment results showing changes in SCN^−^ concentration.

Oxygen amendment	Nutrient amendment	SCN^−^ initial (mg L^−1^)	SCN^−^ final (18 days) (mg L^−1^)
Anoxic	None	131 ± 2.3	132 ± 6.0
	DOC	125 ± 8.8	132 ± 2.0
	PO_4_^3−^	129 ± 3.6	130 ± 9.3
	DOC, PO_4_^3−^	129 ± 5.3	135 ± 2.4
Oxic	None	128 ± 3.1	0.67 ± 1.2
	DOC	126 ± 2.5	5.00 ± 7.8
	PO_4_^3−^	131 ± 3.0	0.00 ± 0.0
	DOC, PO_4_^3−^	123 ± 3.1	0.33 ± 0.6

**Note:**

Errors are shown as 1 standard deviation within triplicate samples of each culture.

### SCN^−^ and NH_4_^+^ biodegradation by a consortium of groundwater microorganisms

**Figure 1:**
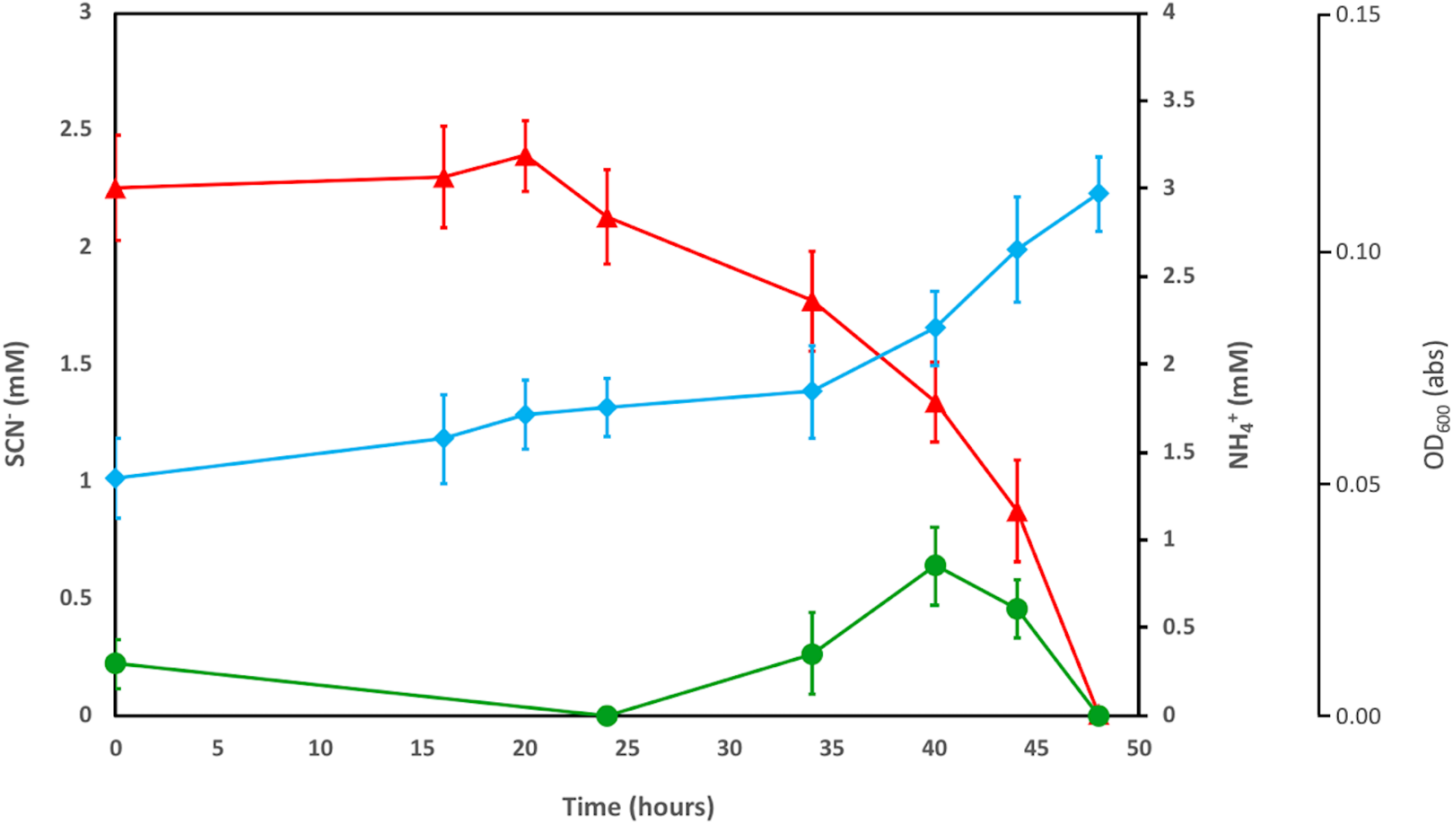
Concentration profiles of SCN^−^ (red) and NH_4_^+^ (green) in SCN^−^ metabolising culturing experiments. The profiles are shown alongside OD_600_measurement (blue) during SCN^−^ removal from filter sterilised groundwater, inoculated with the groundwater culture enriched by addition of DOC and PO_4_^3−^. Error bars are equal to 1 standard deviation within each triplicate.

The enriched microbial consortium, amended with DOC and PO_4_^3−^, was further investigated to determine the fate of the NH_4_^+^ released by SCN^−^ degradation. The consortium completely degraded SCN^−^ in the filter-sterilised groundwater within a period of 50 h (Fig. 1). The initial NH_4_^+^ present in the groundwater was consumed prior to any SCN^−^ removal. After this, an increase in OD_600_ was noted, in tandem with the consumption of SCN^−^ and formation of NH_4_^+^. The concentration of NH_4_^+^ decreased to below detection after all SCN^−^ had been consumed.

### Inhibition of SCN^−^ biodegradation by NH_4_^+^ addition

**Figure 2:**
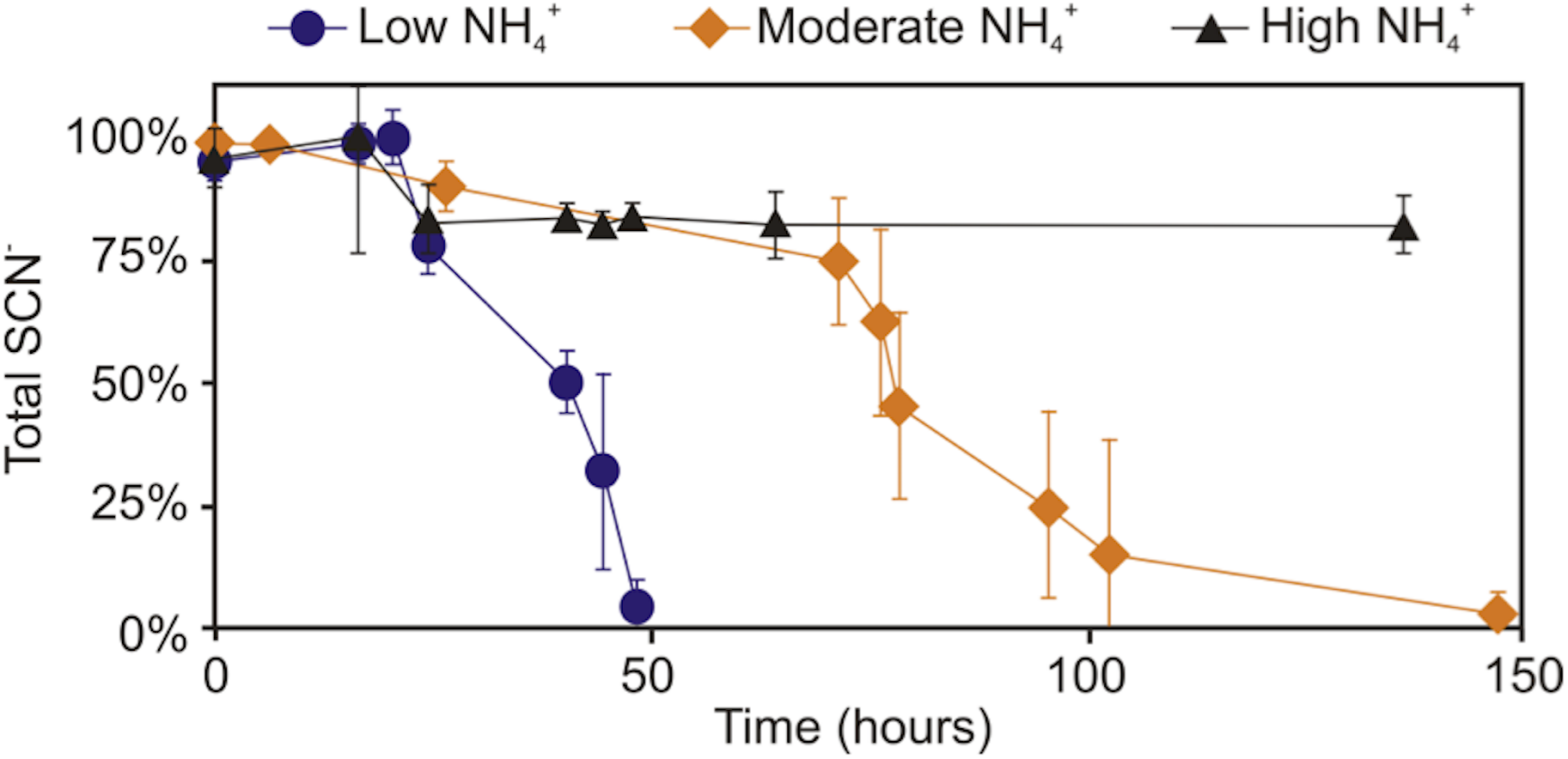
Concentration profile of SCN^−^ and NH_4_^+^ in groundwater SCN^−^ biodegradation culturing experiments. Profiles represent the inoculated (with the DOC and PO_4_^3−^ enriched community) filter sterilised groundwater, in the presence of increasing NH_4_^+^ concentrations. Error bars are equal to 1 standard deviation within triplicates of each experiment.

**Figure 3:**
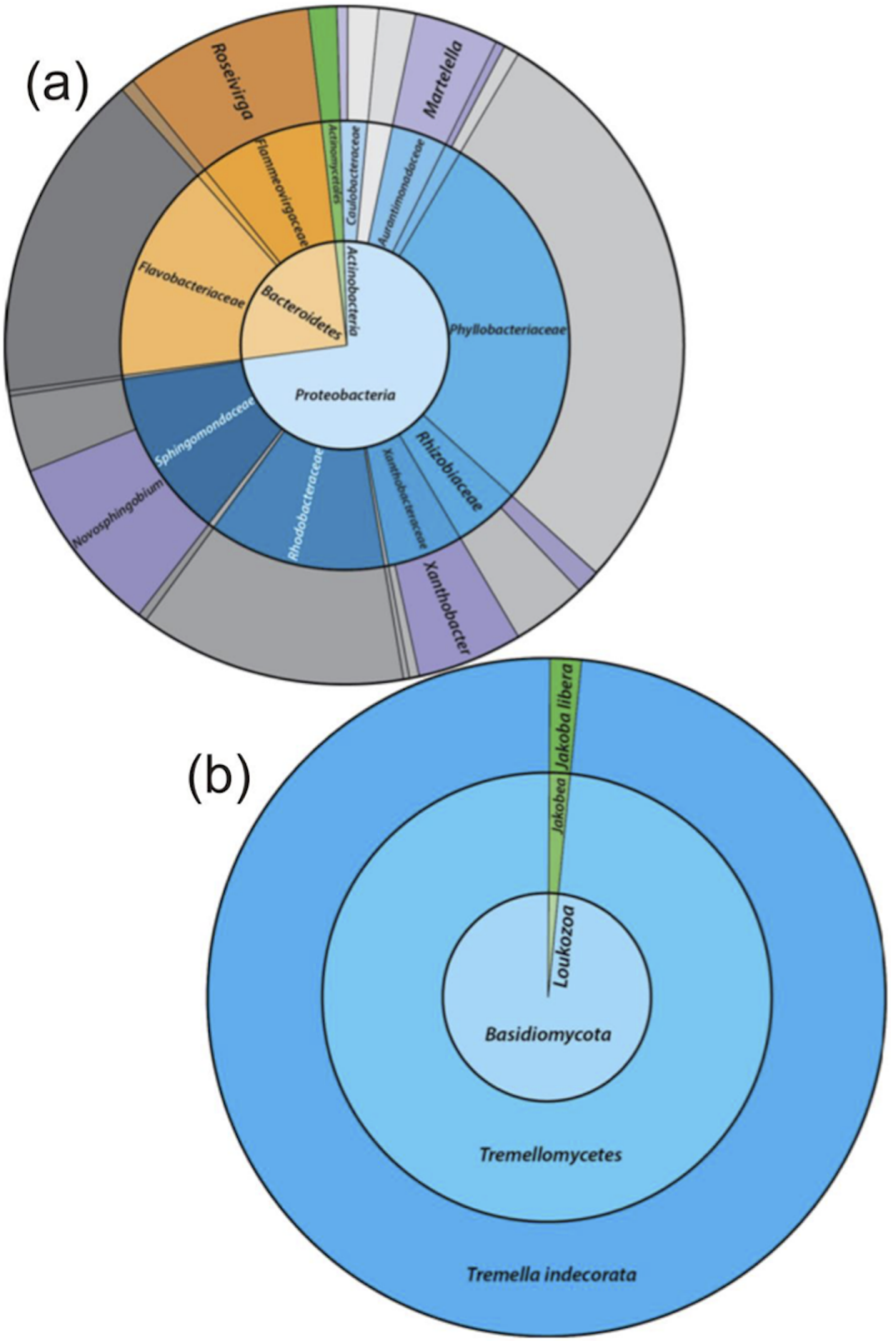
SCN^−^—degradation profiles with increasing NH_4_^+^ concentrations by the enriched microbial consortium. (A) With unamended NH_4_^+^ levels, (B) with moderate NH_4_^+^ levels, and (C) with high NH_4_^+^ levels.

Further experimentation was conducted to determine the effect NH_4_^+^ had upon SCN^−^ biodegradation. This work revealed that low to moderate concentrations of NH_4_^+^ did not inhibit biodegradation of SCN^−^ (Fig. 2). The highest NH_4_^+^ concentration however, completely inhibited SCN^−^ biodegradation. SCN^−^ degradation occurring at lower NH_4_^+^ concentrations only proceeded after complete NH_4_^+^ removal (Fig. 3). Potentially, SCN^−^ biodegradation could have occurred more efficiently after repeated transfers to NH_4_^+^-containing media, but this adaptation hypothesis was not tested in this study.

### Microbial community characterisation by 16S rRNA gene sequencing

**Figure 4:**
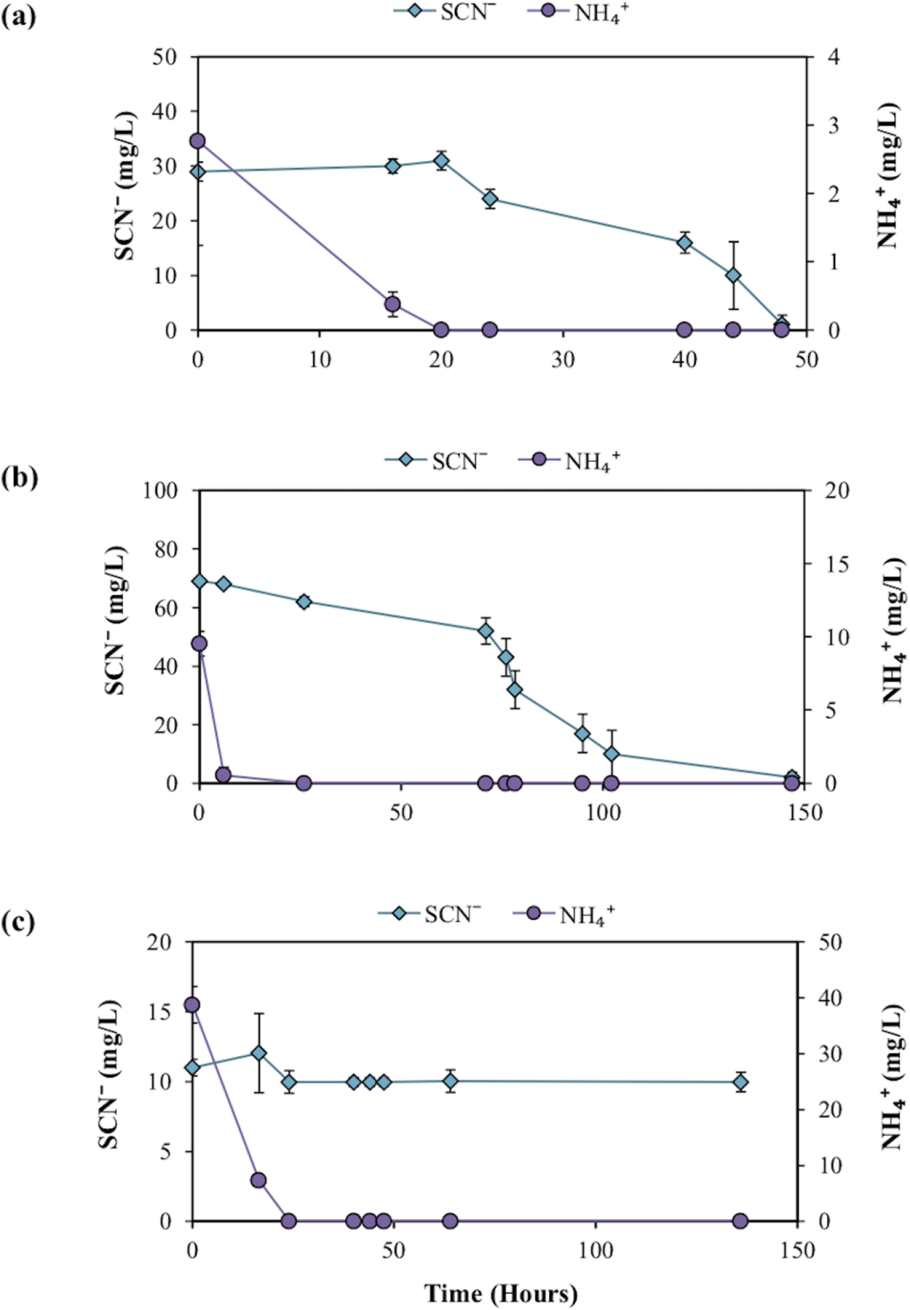
The relative abundance of 16S rRNA gene sequence assignments from the Greengenes database (A) and 18S rRNA gene sequence assignments from the SILVA database (B). Relative abundances are shown for the re-cultured groundwater community amended with DOC and PO_4_^3−^. 16S rRNA gene Taxonomic assignments are defined at the phylum (inner circle), family (middle) and genus (outer) levels, while 18S rRNA gene taxonomic assignments are classified at the phylum (inner), class (middle) and species (outer) levels. Classified taxa comprising ≥1% total abundance are labelled, and grey areas represent unclassified taxa.

The taxonomic assignments for the 16S rRNA gene sequences, from the enriched groundwater community are given in Fig. 4A. At the phylum level, the microbial community enriched through DOC and PO_4_^3−^ addition and exposure to air in the groundwater was dominated by *Proteobacteria* (72.8%) and *Bacteroidetes* (25.8%), with a minor proportion of *Actinobacteria* (1.3%). The dominant families within the *Proteobacteria* were *Phyllobacteriaceae* (27.8%), *Rhodobacteriaceae* (12.8%) and *Sphingomonadaceae* (12.5%). The latter was entirely assigned to the *Novosphingobium* genus, and the dominant OTU found to be most closely related to *Novosphingobium panipatense* strain UMTKB-4 (99%), by comparison to NCBI and Greengenes databases (Tables A4 and A5). The *Phyllobacteriaceae* family was dominated by a single unclassified OTU (27.8%), most closely related to an uncultured *Mesorhizobium* sp. clone S3_F08 (99% similarity). The two dominant OTUs for the *Rhodobacteraceae* family were most closely related to an uncultured bacterium clone MAL_E01 (12.8% abundance, 99% similarity), the higher abundance of the two OTUs had equal sequence similarity to an environmental sample, *Thioclava pacifica* (98% similarity) known to be capable of sulphur oxidation and consumption of simple organics (Sorokin et al., 2005). In addition to these high abundance members, sequences assigned to the genera *Martelella* sp. (4.0%) and *Xanthobacter* (5.4%) made up significant minority taxa.

The *Bacteroidetes* phylum was largely dominated by a single OTU unassigned using the Greengenes database below family level, but most closely related to an uncultured *Owenweeksia* sp. Clone (99% similarity). Other *Bacteroidetes* sequences assigned to the *Flammeovirgaceae* family belong to the *Roseivirga* genus.

### Microbial community characterisation by 18S rRNA gene sequencing

The 18S rRNA gene analysis showed a simple eukaryotic distribution (Fig. 4B), with only two unique OTUs identified: *Tremella indecorata* (98.5%), a fungus of the *Basidiomycota* phylum and *Jakoba libera* (1.5%), a trophic flagellate from the *Loukozoa* phylum. The SILVA assigned taxonomies for these OTUs were compared to the identities assigned by the NCBI database by BLAST (Table A5). The dominant OTU, *Tremella indecorata* was most closely related as *Tremellales* sp. LM630 (99% similarity), while the less abundant OTU was most closely related to *J. libera* at 99% gene sequence similarity, which is consistent with the SILVA identity.

## Discussion

The microbial consortium enriched from SCN^−^ contaminated groundwater was able to consume SCN^−^ aerobically in the presence of all nutrient amendments; however, under anoxic conditions, no SCN^−^ degradation was observed. This dependence on oxygen (present as air) supports the interpretation that SCN^−^ degradation progressed via aerobic respiration. In fact, anoxic pathways have not been observed, with a notable exception through coupling to nitrate or nitrite reduction (Sorokin et al., 2004). Significantly, only the culture amended with both DOC and PO_4_^3−^ resulted in a microbial consortium capable of consistently performing SCN^−^ degradation. This suggests that in situ SCN^−^ degradation in groundwater by native SCN^−^ degrading microorganisms requires addition of both of these nutrients for sustained contaminant removal. We acknowledge, however, that the composition and performance of groundwater-derived SCN^−^ degrading microbial consortia may vary significantly with temperature. If so, heating of the groundwater to 20–30 °C, either in situ or ex situ, may also be required to achieve results similar to those presented here for incubated culturing experiments.

Both autotrophic and heterotrophic SCN^−^-degrading organisms are known to assimilate the NH_4_^+^ released from SCN^−^ degradation as their sole source of nitrogen (Stafford & Callely, 1969). The presence of NH_4_^+^ likely represented a preferential source of nitrogen, in comparison to SCN^−^, thereby potentially inhibiting degradation (Stafford & Callely, 1969). In the absence of added NH_4_^+^, SCN^−^ -derived NH_4_^+^ was removed, potentially as a N requirement for SCN^−^-degraders or other microbial community members. The amount of N released during SCN^−^ metabolism was on the order of one mM. Typically, pure cultures of known SCN^−^ oxidisers that derive their N solely from SCN^−^ are grown on ∼1.5 mM N (e.g. DSMZ *Thiobacillus* medium). So the amount of N provided by SCN^−^ metabolism in our experiments was roughly 66% that provided in pure culture media, which seems sufficient to stimulate cell growth. In the NH_4_^+^—amended cultures, we provided ∼160 μM, 500 μM and two mM N for low, moderate and high NH_4_^+^ amendments, respectively. Significantly, the consumption of NH_4_^+^ indicated that the consortium was able to circumvent complete inhibition of SCN^−^ biodegradation at higher NH_4_^+^ concentrations. Although NH_4_^+^ removal through oxidation is also a possibility, no known autotrophic bacteria or archaea widely responsible for this metabolic trait were identified in this microbial community.

As the consortium may have utilised SCN^−^ as a source of energy, sulphur, nitrogen or carbon (Gould et al., 2012), a number of metabolic niches might be associated with its degradation and the cycling of the released nutrients. A limitation of our study is the lack of data for the biodegradation products of SCN^−^ or glucose that might have allowed for a better understanding of SCN^−^ metabolism. Certain intermediate metabolites that are diagnostic of which pathway is utilised in SCN^−^ metabolism may also have been lost to volatilisation, for example, carbonyl sulphide. The 16S rRNA gene sequencing identified few taxa associated with SCN^−^-degradation, the most abundant being an OTU (∼4.0%) assigned to the *Sphingomonadaceae* family, specifically the *Sphingopyxis* and *Sphingomonas* genera (Du Plessis et al., 2001; Felföldi et al., 2010).

The consortium was found to include dominant genera that share significant sequence similarity to a known sulphur-oxidising genus, *Thioclava* sp., which has demonstrated chemoautotrophic growth on intermediate sulphur compounds including thiosulphate, and heterotrophic growth on simple organics including glucose (Sorokin et al., 2005). We note that certain *Thioclava* species may consume NH_4_^+^ as a source of nitrogen (Sorokin et al., 2005). Furthermore, nitrogen-fixing bacteria were also represented in the community: *Novosphingobium* (Kaneko et al., 2000), potentially having a role in nitrogen supply when NH_4_^+^ was absent.

The cultured microbial consortium was dominated by heterotrophs that likely played an important role in the cycling of carbon and possibly nitrogen. *Martelella* sp., *Thioclava* sp., *Novosphingobium* sp., *Roseivirga* sp. and *Basidiomycota* are all known to consume various forms of organic carbon, including glucose (Chung et al., 2016; Sorokin et al., 2005; Chen et al., 2015; Nedashkovskaya et al., 2008; Prillinger & Lopandic, 2015). The dominance of heterotrophs in this consortium suggests that the SCN^−^ may mostly have been degraded by heterotrophs as a source of nitrogen (via the released NH_4_^+^), rather than by autotrophs utilising sulphur oxidation as an energy source. This interpretation can be compared against previously documented communities dominated by autotrophic SCN^−^-degraders often belonging to the *Thiobacillus* genus (Felföldi et al., 2010; Huddy et al., 2015; Kantor et al., 2015, 2017; Watts et al., 2017). We acknowledge, however, that because neither background DOC nor amended glucose were measured before or during our experiments, we can only speculate on the relationship among SCN^−^ degradation, glucose consumption and nitrogen assimilation.

The importance of heterotrophs in the SCN^−^-degrading consortium is not well understood, with only *Sphingomonadaceae* family associated with bacterial SCN^−^-degradation. Their ability to prevent the accumulation of the inhibitor NH_4_^+^, likely through assimilation, may be significant when considering the implementation of a bioremediation strategy. Previous SCN^−^-degrading communities have also been shown to be incapable of preventing NH_4_^+^ accumulation from SCN^−^ degradation (Shoji et al., 2014), while other approaches have coupled SCN^−^-biodegradation to nitrification/denitrification (Villemur et al., 2015) or assimilation to biomass by algae (Ryu et al., 2014). We note that, to date, only the *Acremonium* and *Fusarium* genera contain species known to degrade SCN^−^ (Kwon, Woo & Park, 2002; Medina et al., 2012). Regardless, the coupling of SCN^−^-biodegradation with microbial NH_4_^+^-removal is an important requirement to perform the complete bioremediation of SCN^−^ and its potential intermediate degradation products.

## Conclusion

The results presented here demonstrate that naturally occurring SCN^−^-degrading microbial consortia could be enriched and directly stimulated from SCN^−^ contaminated groundwater. The promotion of this extant microbial community, already adapted to the presence of SCN^−^ and the prevailing groundwater chemistry, would preclude the need to bio-engineer externally a mixed community or pure culture, which may be illsuited to these conditions. Interestingly, unlike other reported SCN^−^-degrading bioreactor communities (Huddy et al., 2015, Ryu et al., 2015, Kantor et al., 2015; Watts et al., 2017), our consortium did not contain significant populations of *Thiobacilli*, previously implicated as the principle SCN^−^-degraders.

When considering in situ SCN^−^ bioremediation solutions, the lack of oxygen in the groundwater appears to be the most important inhibitor of SCN^−^ biodegradation. Exposing contaminated groundwater to air may therefore stimulate SCN^−^ biodegradation, a significant finding considering that most TSFs are unlined and therefore result in seepage of SCN^−^ through to poorly-oxygenated groundwater. This fact suggests in situ natural attenuation may be an oxygen-limited process, with implications for the design of a bioremediation strategy involving both nutrient and air amendments in a controlled bioreactor containing SCN^−^-degrading microorganisms sourced from locally contaminated groundwater.

## Supplemental Information

10.7717/peerj.6498/supp-1 Supplemental Information 1 Spurr et al. Excel raw data. Raw data spreadsheet for Spurr et al.

10.7717/peerj.6498/supp-2 Supplemental Information 2 Additional water quality data for groundwater samples.

10.7717/peerj.6498/supp-3 Supplemental Information 3 OTU identity comparison for 16S rRNA sequences between BLAST and GreenGenes classification.

10.7717/peerj.6498/supp-4 Supplemental Information 4 OTU identity comparison for 16S rRNA sequences between BLAST and GreenGenes classification.

10.7717/peerj.6498/supp-5 Supplemental Information 5 Table A4: OTU closest match (99%) for 16S rRNA gene sequences (prokaryotes)/ Table A5: OTU closest match (99%) for 16S rRNA gene sequences (eukaryotes).

## Additional Information and Declarations

### Data Availability

The following information was supplied regarding data availability: Raw data for all tables and figures are available in the Supplemental Materials.
