# Supplementary material for: Biodegradation of thiocyanate by a native groundwater microbial consortium
Source: PeerJ. 2019 Mar 26;7:e6498. doi: 10.7717/peerj.6498 (PMC6440457; doi:10.7717/peerj.6498)
Supplement: Supplemental Information 3 [file peerj-07-6498-s003.docx]

**Table A2.** OTU identity comparison for 16S rRNA sequences between BLAST and GreenGenes classification.

| **Sequence similarity** | **BLAST Identity** | **GenBank Sequence ID** | **GreenGenes Identity** |
| --- | --- | --- | --- |
| 99% | Uncultured *Mesorhizobium* sp. clone S3_F08 | KP182007.1 | *Phyllobacteriaceae* (family) |
| 99% | Uncultured *Owenweeksia* sp. clone | JX530590.1 | *Flavobacteriaceae* (family) |
| 99% | Uncultured bacterium clone MAL_E01 (#2 on list: 98% Thioclava sp.) | KR921275.1 | *Rhodobacteriaceae* (family) |
| 99% | *Roseivirga* sp. D-25 | KM587636.1 | *Roseivirga* sp. |
| 99% | *Novosphingobium* *panipatense* strain UMTKB-4 | KT025847.1 | *Novosphingbium* sp. |
| 99% | Uncultured bacterium clone nbw390g07c1 | GQ096648.1 | Sphingomonadaceae (family) |
| 99% | *Martelella* sp. YC7034 | KR233160.1 | *Martelella* sp. |
| 99% | *Martelella* *mediterranea* strain NJES-108 | KR140271.1 | *Rhizobiaceae* (family) |
| 99% | Uncultured bacterium clone 0010Ak1_E2 | KF558803.1 | *Martelella* sp. |
| 99% | Uncultured *rhodospirillales* bacterium clone KF130_10F01 | EU361456.1 | *Kiloniellales* (order) |
| 99% | *Xanthobacter* sp. LAA-2009-i49 strain i49 | FN298500.1 | *Xanthobacter* *autotrophicus (sp.)* |
| 99% | Uncultured bacterium clone HK34-1-10-1 | KX163485.1 | *Caulobacteriaceae* (family) |
| 99% | *Xanthobacter* *Flavus* strain LEM28 | KU180350.1 | *Hyphomicrobaceae* (family) |
