# Supplementary material for: Biodegradation of thiocyanate by a native groundwater microbial consortium
Source: PeerJ. 2019 Mar 26;7:e6498. doi: 10.7717/peerj.6498 (PMC6440457; doi:10.7717/peerj.6498)
Supplement: Supplemental Information 4 [file peerj-07-6498-s004.docx]

**Table A3.** OTU identity comparison for 16S rRNA sequences between BLAST and GreenGenes classification.

| **Sequence similarity** | **BLAST Identity** | **GenBank Sequence ID** | **SILVA Identity** |
| --- | --- | --- | --- |
| 99% | *Jakoba* *libera* | AY117418.1 | *Jakoba* *libera* (sp.) |
| 99% | *Tremellales* sp. (LM630 strain) | EF060914.1 | *Tremella* *indecorata* (sp.) |
