# Supplementary material for: Biodegradation of thiocyanate by a native groundwater microbial consortium
Source: PeerJ. 2019 Mar 26;7:e6498. doi: 10.7717/peerj.6498 (PMC6440457; doi:10.7717/peerj.6498)
Supplement: Supplemental Information 5 [file peerj-07-6498-s005.docx]

**Table A4.** OTU closest match (99%) for 16S rRNA gene sequences (prokaryotes).

**Table A5.** OTU closest match (99%) for 16S rRNA gene sequences (eukaryotes).
